# Parkinsonian Syndrome Diagnosed via Novel Alpha-Synuclein Skin Biopsy in a Patient Presenting With Catatonic Symptoms

**DOI:** 10.7759/cureus.73441

**Published:** 2024-11-11

**Authors:** Nicolas Biaggi, Alfred Torres, Melissa L Verzura, Jose Cruz, Rogelio Suarez

**Affiliations:** 1 Psychiatry, Mount Sinai Medical Center, Miami Beach, USA; 2 Medical School, Florida International University, Herbert Wertheim College of Medicine, Miami, USA; 3 Psychiatry/Neurology, Mount Sinai Medical Center, Miami Beach, USA

**Keywords:** alpha-synuclein biopsy, bradykinesia, catatonia, major depressive disorder (mdd), parkinson-plus syndromes, parkinson's disease, psychomotor retardation

## Abstract

The differential diagnosis of neurocognitive and psychiatric disorders, particularly when symptoms overlap significantly, poses a substantial challenge in clinical practice. Parkinson’s disease (PD), Lewy body dementia, and catatonia are distinct conditions that can present with similar motor and cognitive symptoms, complicating accurate diagnosis and effective treatment.

We report the case of a 45-year-old male patient who presented for electroconvulsive therapy (ECT) evaluation. He was initially diagnosed with major depressive disorder with catatonic features. The clinical overlap between psychomotor retardation due to severe depression and bradykinesia, characteristic of PD, led to a reevaluation of the initial diagnosis. A novel diagnostic tool, the alpha-synuclein skin biopsy, was utilized and it revealed the presence of alpha-synuclein pathology. This ultimately led to the diagnosis of parkinsonian syndrome.

The positive alpha-synuclein biopsy result was pivotal in distinguishing between catatonia and parkinsonian syndrome, facilitating the initiation of appropriate treatment. The patient’s subsequent improvement underscores the importance of considering neurodegenerative etiologies in patients with atypical or treatment-resistant psychiatric symptoms.

This case underscores the need for a comprehensive, multidisciplinary approach to evaluating patients with overlapping psychiatric and neurological symptoms. Integrating novel diagnostic tools, such as the alpha-synuclein biopsy, into clinical practice may enhance diagnostic accuracy and improve patient outcomes.

## Introduction

The differential diagnosis of neurocognitive and psychiatric disorders is a complex and often challenging task, particularly when symptoms overlap significantly. Diagnostic intricacies may be faced when distinguishing between Parkinson’s disease (PD), Lewy body dementia (LBD), and catatonia. Each of these conditions can present with a range of symptoms that might obscure their true etiology, complicating diagnosis and treatment. PD, recognized for its motor symptoms such as tremors and rigidity, can manifest cognitive impairment that mimics symptoms seen in dementia. The motor symptoms may also overlap with other disease processes as movements may be slower and patients may have trouble initiating movements in general [1]. LBD is characterized by progressive cognitive decline and changes in behavior accompanied by postural instability and bradykinesia [2]. LBD, along with multiple system atrophy and progressive supranuclear palsy are some of the more common disorders that have been characterized as Parkinsonism plus syndrome [3]. Catatonia, though less commonly associated with neurodegenerative processes, can present with severe motor disturbances and altered consciousness. It is a syndrome characterized by a range of symptoms including immobility, stupor, and bizarre postures that may be associated with psychiatric or neurological disorders. Some overlapping movement symptoms include lead-pipe rigidity and lack of purposeful movement [4]. Although the disorders listed may have varying diagnostic criteria and key features, the intricacies living within gray areas may lead to confusion and error in diagnosis. Utilizing diagnostic tools such as the novel alpha-synuclein skin biopsy test, clinical assessments, and diagnostic criteria can help distinguish PD from catatonia and other synucleinopathies and guide treatment, leading to more optimal outcomes. Alpha-synuclein goes through different post-translational modifications, causing it to aggregate and contribute to disease pathogenesis. The alpha-synuclein protein starts accumulating in the neurons of the colon, skin, genitourinary tract, salivary glands, and the heart during the early phase [5]. The skin is the easiest organ to access, and hence, is an accessible place to detect and quantify alpha-synuclein deposits. This test is a minimally invasive procedure involving a skin punch biopsy from the neck, back of the knee, or ankle. The sensitivity of this test may be altered by the depth and location of the biopsy although it is quite reliable and consistently above 80% [5]. Understanding the similarities across various conditions is crucial for clinicians to avoid misdiagnosis and ensure that patients receive appropriate care tailored to their specific needs [6]. The information listed above underscores the importance of a comprehensive diagnostic approach. We present a case that was referred to our office at Mount Sinai Medical Center for evaluation of a catatonia diagnosis, but eventually diagnosed with parkinsonian syndrome after alpha-synuclein was detected upon skin biopsy.

## Case presentation

A 45-year-old male patient presented to the Mount Sinai Medical Center Behavioral Health Department with a psychiatric diagnosis of major depressive disorder with catatonic features, and generalized anxiety disorder. He was referred for electroconvulsive therapy (ECT) evaluation. He was accompanied by his mother who provided collateral information.

The patient had lived a regular life, finished high school, and started working in the music industry. He was divorced and had a 15-year-old daughter. He reported that his symptoms had started three and a half years before this evaluation. His first symptom was described as “slowed body movements” that started interfering with his job. He thus sought care and was referred to a psychiatrist who diagnosed him with "depression and anxiety." He was treated by the psychiatrist for around seven months with multiple medications that he did not recall, but he did not show any improvement. Due to co-existing cannabis use, he decided to attend a dual diagnosis rehab center. He stated that he was given “over 15 medications” there (which he did not recall the names of) but did not obtain any symptomatic relief. He reported seeing other doctors at that time, including a neurologist, and reported normal workups. He then decided to move into his mother’s house and started attending college again. He reported difficulty finding a job due to the way he appeared. He started seeing a different psychiatrist, who diagnosed him with major depressive disorder with catatonic features. After one and a half years of treatment with multiple medications and 30 sessions of Transcranial Magnetic Stimulation (TMS), he was recommended an evaluation for ECT.

On the day of the initial evaluation, the patient was being managed with bupropion XL 300 mg daily, mirtazapine 7.5 mg nightly, and lorazepam 1 mg three times daily. At that time, he reported vague symptoms of depression, which he characterized as low energy, episodes of sadness, and lack of concentration. He also reported anxiety, described as excessive worries and tensions, which he attributed to driving long distances. He reported hallucinations, occasionally auditory, but mainly visual. He described them as shadows, wavy lines on tiles, sometimes seeing other people in the house, or feeling that his sister was standing behind him. His sleep was disturbed along with an inability to fall asleep. He also presented with subjective short-term memory loss. His mom stated he was repeating questions and forgetting to close doors at times. He was neatly dressed and spoke in a soft, slow, and monotonous tone. He had poor eye contact with slowed thinking and thought blocking. He exhibited a stooped posture, walked with a shuffling gait, and had slowed movements. He endorsed he had trouble initiating movements, even freezing up sometimes, and stated he felt as if “[his] feet were made of lead.” He also exhibited micrographia and cogwheel rigidity. His Mini-Mental State Examination (MMSE) score was 23/30 during this evaluation. The patient lost one point in orientation as he was unable to state the date, lost five points in attention as he was unable to perform serial sevens, and then lost one point in recall. He had a Bush Francis catatonia rating scale score of four.

After this visit, he was recommended to continue the same medications and follow up with the Wein Center for evaluation of possible neurodegenerative disease before starting ECT.

**Figure 1:**
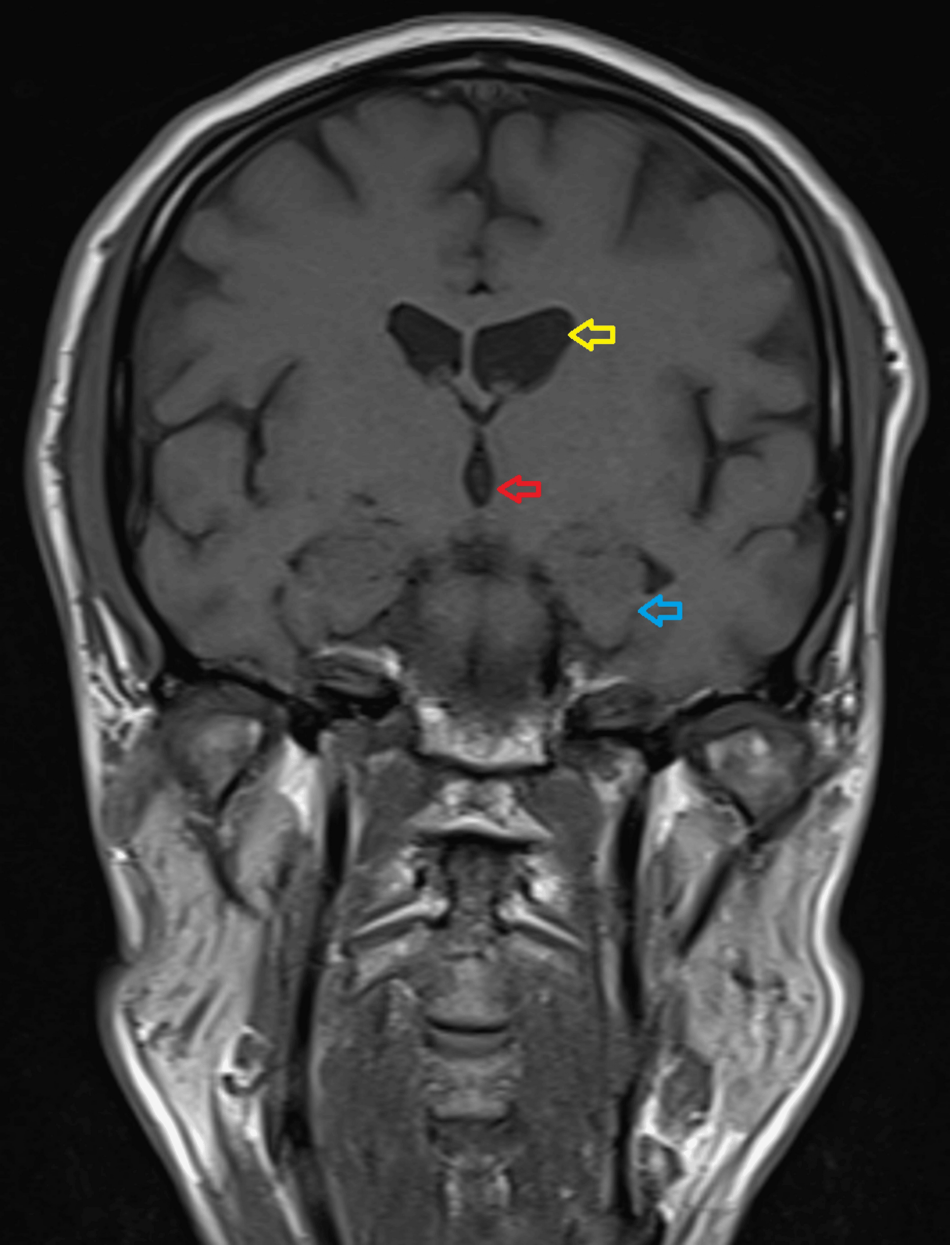
Coronal brain MRI showing the mild enlargement of the lateral ventricles (yellow arrow), minimal enlargement of the third ventricle (red arrow), and normal hippocampus volume (blue arrow)

**Figure 2:**
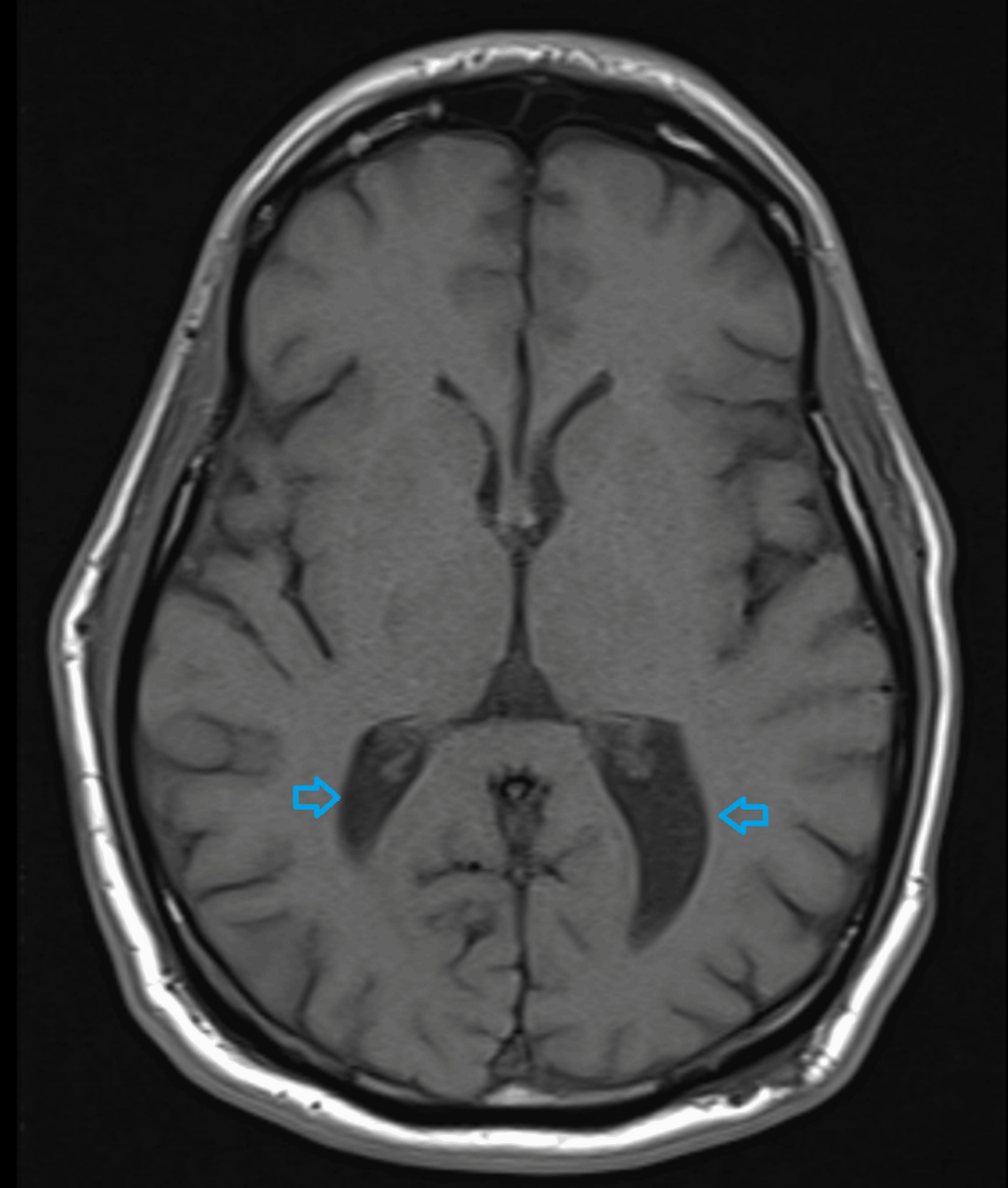
Axial brain MRI showing the mild enlargement of the occipital horn of the lateral ventricles (blue arrows)

At the Wien Center evaluation, which occurred four weeks after the initial psychiatric evaluation, the patient showed symptoms of “catatonia” included posturing, poor eye contact, rigidity, and slowed movements. Once again, he had a Bush Francis score of four. His speech was non-spontaneous but he was able to reply in a soft tone and low volume when spoken to. An MRI revealed no significant generalized atrophy, mild enlargement of the occipital horn of the lateral ventricles (L>R), mild enlargement of the lateral ventricles, minimal enlargement of the third ventricle, and normal hippocampus volume (Figures 1, 2).

The patient reported that he was taking lorazepam as needed and had not noticed any improvement in his symptoms. He was recommended to continue his medications and take lorazepam as scheduled. An alpha-synuclein biopsy was planned.

Six weeks later, the patient was seen again via telehealth. He was accompanied by his mother. They reported that his movements were worsening, and were characterized by bradykinesia and posturing. They stated that he "seems frozen, [he] cannot get out of bed." He continued taking lorazepam, but did not improve. He reported increased depression due to the lack of improvement. The family stated they would like him to undergo the alpha-synuclein skin biopsy before proceeding to ECT for catatonia. The lorazepam dosage was also increased to 2 mg three times daily to target possible catatonia.

The patient returned five days later for a skin biopsy. At the time of evaluation, he reported feeling “more tired,” likely due to the increased lorazepam dose. But he still reported no improvement despite this adjustment. He continued having difficulty getting out of bed, bradykinesia, and poor eye contact. The alpha-synuclein biopsy was performed and yielded a positive result. The patient was then recommended to follow up with neurology.

Two months later, the patient reported that after his neurology follow-up, he was started on carbidopa/levodopa 25/100 mg three times daily for his motor dysfunction. He reported significant improvement in bradykinesia and described feeling "smoother, not as rigid." Pimavanserin 34 mg daily was also introduced to address his psychotic symptoms, leading to a moderate reduction in hallucinations. The amelioration of his motor symptoms corresponded with an alleviation of his affective disturbances, and his mild anxiety was subsequently managed by his primary psychiatrist.

## Discussion

This case presents a 45-year-old male patient who was initially diagnosed with major depressive disorder with catatonic features. The diagnosis was modified to parkinsonian syndrome following an alpha-synuclein skin biopsy. The patient’s initial presentation included both motor and psychiatric symptoms, making it challenging to differentiate and diagnose him. This also highlights the complexity of these cases where psychiatric and motor conditions overlap.

Diagnostic challenges

The patient’s most prominent symptoms were motor in nature. This brings in the first challenge of bradykinesia, catatonia, and psychomotor retardation. Psychomotor retardation, described in the literature, emphasizes disturbances in speech, facial expression, fine motor behavior, gross locomotor activity, or ideation [7]. Bradykinesia, a component of psychomotor retardation, can be described by the slowness of performed movements, poverty of spontaneous movements or associated movements, freezing, or prolonged time taken to initiate a movement. These movements can also be smaller than desired, as seen in micrographia [8]. The movements described in bradykinesia are a key element of PD [9]. Like bradykinesia, catatonia can present with psychomotor retardation, where the patient exhibits slowed movements, decreased speech, and reduced responsiveness [10].

The patient’s symptoms, particularly the slowed movements and cognitive disturbances, could have been easily misattributed solely to a psychiatric disorder. The diagnostic challenge was further compounded by the patient's lack of improvement with standard psychiatric treatments, including a combination of antidepressants, benzodiazepines, and TMS. The clinical overlap between psychomotor retardation associated with severe depression and bradykinesia due to an underlying neurological condition posed a significant challenge. The persistence of symptoms despite psychiatric interventions raised the suspicion of an underlying neurological condition. Furthermore, during our evaluation, the presence of prominent symptoms of bradykinesia led to further evaluation before ECT.

Role of alpha-synuclein biopsy

The turning point in this case was the positive alpha-synuclein biopsy, which led to the diagnosis of a parkinsonian syndrome, likely a form of Parkinson plus syndrome. Parkinson plus syndromes, such as Dementia with Lewy Bodies (DLB), Multiple System Atrophy (MSA), Progressive Supranuclear Palsy (PSP), and Corticobasal Degeneration (CBD), involve parkinsonian symptoms along with additional neurological features [11]. Further clinical evaluation and monitoring, though, are required to reach a definitive diagnosis. Still, due to the positive alpha-synuclein biopsy result, treatment and management was tailored for better results in this patient.

## Conclusions

The intricacy of this case emphasizes the need for a multidisciplinary approach in diagnosing and managing patients with overlapping psychiatric and neurological symptoms. The eventual diagnosis of a neurodegenerative disorder, despite initial delays, highlights the necessity for comprehensive neurological evaluations in patients presenting with treatment-resistant psychiatric symptoms and atypical motor findings. Utilizing innovative and novel diagnostic tools like the alpha-synuclein biopsy can offer valuable insights and inform appropriate treatment plans. It also emphasizes the need to consider neurodegenerative disorders in patients exhibiting atypical or resistant psychiatric symptoms, advocating for a thorough and collaborative approach to patient care.
